# Experimental Investigation of Stability of Silica Nanoparticles at Reservoir Conditions for Enhanced Oil-Recovery Applications

**DOI:** 10.3390/nano10081522

**Published:** 2020-08-04

**Authors:** Shidong Li, Yeap Hung Ng, Hon Chung Lau, Ole Torsæter, Ludger P. Stubbs

**Affiliations:** 1 Institute of Chemical and Engineering Sciences, Agency for Science, Technology and Research (A*STAR), Singapore 627833, Singapore; ng_yeap_hung@ices.a-star.edu.sg (Y.H.N.); ceelhc@nus.edu.sg (H.C.L.); ludger_paul@ices.a-star.edu.sg (L.P.S.); 2 Department of Civil and Environmental Engineering, National University of Singapore, Singapore 117576, Singapore; 3 PoreLab, Norwegian Center of Excellence, Norwegian University of Science and Technology (NTNU), 7031 Trondheim, Norway; ole.torsater@ntnu.no; 4 Department of Geoscience and Petroleum, Norwegian University of Science and Technology (NTNU), 7031 Trondheim, Norway

**Keywords:** nanoparticle stability, reservoir condition, reservoir rock, crude oil, nanoparticle agglomeration

## Abstract

To be effective enhanced oil-recovery (EOR) agents, nanoparticles must be stable and be transported through a reservoir. However, the stability of a nanoparticle suspension at reservoir salinity and temperature is still a challenge and how it is affected by reservoir rocks and crude oils is not well understood. In this work, for the first time, the effect of several nanoparticle treatment approaches on the stability of silica nanoparticles at reservoir conditions (in the presence of reservoir rock and crude oil) was investigated for EOR applications. The stability of nanoparticle suspensions was screened in test tubes at 70 °C and 3.8 wt. % NaCl in the presence of reservoir rock and crude oil. Fumed silica nanoparticles in suspension with hydrochloric acid (HCl), polymer-modified fumed nanoparticles and amide-functionalized silica colloidal nanoparticles were studied. The size and pH of nanoparticle suspension in contact with rock samples were measured to determine the mechanism for stabilization or destabilization of nanoparticles. A turbidity scanner was used to quantify the stability of the nanoparticle suspension. Results showed that both HCl and polymer surface modification can improve nanoparticle stability under synthetic seawater salinity and 70 °C. Suspensions of polymer-modified nanoparticles were stable for months. It was found that pH is a key parameter influencing nanoparticle stability. Rock samples containing carbonate minerals destabilized unmodified nanoparticles. Crude oil had limited effect on nanoparticle stability. Some components of crude oil migrated into the aqueous phase consisting of amide-functionalized silica colloidal nanoparticles suspension. Nanoparticles modification or/and stabilizer are necessary for nanoparticle EOR application.

## 1. Introduction

In recent years, nanotechnology research on enhanced oil recovery (EOR) has shown promising results in the laboratory. Some EOR experiments with silica nanoparticles have been performed and showed positive results in increasing oil recovery [1,2,3,4]. The proposed EOR mechanisms for silica nanoparticles include interfacial tension reduction, wettability alteration, plugging of pore channels, disjoining pressure and emulsification [5,6,7,8].

The prerequisite of these mechanisms working well in the reservoir is that nanoparticles are stable at reservoir conditions so that they can maintain their surface activities for EOR. The nanofluid preparation method is crucial for its stability. For EOR applications, the most commonly used base fluid is brine. In the stationary state, the settling velocity of small spherical particles in a suspension follows Stokes law [9]:

(1) $$V=\frac{2R^{2}}{9\mu }\left(\rho_{p}-\rho_{L}\right)\cdot g$$

where *V* is the particle’s settling velocity; *R* is the spherical particle’s radius; µ is the liquid medium viscosity; *ρ_p_* and *ρ_L_* are the particle and the liquid medium density, respectively, and *g* is the acceleration of gravity. This equation explains the effect of gravity, buoyancy and viscous drag on the behavior of the suspended particles in the base fluid. According to Stokes law, the nanoparticle settling velocity decreases with: (1) reducing *R*, (2) increasing *µ*, the base fluid viscosity and (3) lessening the density difference between nanoparticles and base fluid [10].

There are two different techniques used to produce nanofluids. First, a two-step technique in which a dry nanoparticle powder is first produced and then dispersed in a base fluid. However, due to the high surface energy of nanoparticles, aggregation and clustering easily take place after preparation. Therefore, additional techniques are needed to minimize this problem, such as a high shear homogenization and ultrasound. Alternatively, a single-step technique can be applied wherein nanoparticles synthesis and nanofluid preparation are undertaken simultaneously. In the single-step method, there is no drying, storage, transportation, and dispersion of nanoparticles, so the aggregation of nanoparticles is minimized and the stability of the nanofluid can be improved [10].

However, due to van der Waals attraction between nanoparticles and high-temperature and high-salinity conditions in a reservoir, the injected nanoparticles may still undergo aggregation and agglomeration, thus losing their colloidal stability. In order to maintain long-term stability of a nanofluid in a reservoir, some physical or/and chemical treatments are necessary [10]. Generally, nanoparticles have to meet two principles to achieve good stability. The first is the diffusion principle: The nanoparticles are scattered by and dispersed into a liquid medium. The second is the Zeta potential principle: the absolute zeta potential value of a nanofluid must be larger than a specified value and have a sufficient repulsive force between the nanoparticles [11]. The commonly used techniques to increase nanofluid stability are summarized as follows. The first technique is pH control. The stability of a nanofluid is directly related to its electrokinetic properties. Strong repulsion forces can create a stable nanofluid through a high surface charge density [12,13,14]. Sofla et al. proposed “H^+^ protection” theory. They showed that adding hydrochloric acid (HCl) into a nanofluid can effectively stabilize silica nanoparticles in seawater [15]. The second technique is addition of a surfactant. This is a general method used to improve the stability of nanoparticles in aqueous phase. The mechanism is that the hydrophobic surface of nanoparticles is covered with surfactant and changed to hydrophilic, thus the repulsion force will become larger and absolute value of zeta potential will increase [16,17]. Surfactant selection (cationic, anionic or non-ionic) is very important [18]. The third technique is surface modification (steric stabilization). This method utilizes the adsorption of large molecules like surfactants and polymers on the surface of nanoparticles to prevent aggregation. The adsorbed molecules lead to an increase of osmotic repulsion, resulting in higher colloidal stability of the nanofluid [19]. Ranka et al. achieved a stable nanofluid by modifying silica nanoparticles with zwitterionic polymers [20]. The fourth method is ultrasonication to break down the agglomeration and clustering of nanoparticles [21]. Therefore, particle size will be reduced remarkably.

After a nanofluid is prepared by using the aforementioned techniques, specific analytical methods are needed to evaluate the relative stability of the nanofluid samples:

(1) Optical visualization. This is a simple method to determine the stability of nanofluids in test tubes by optical inspection for sedimentation after certain periods of time [10]. This method is only applicable for low viscosity and transparent base fluids.

(2) Zeta potential measurement. This method is one of the most critical tests to validate the stability of nanofluids. If the zeta potential has a high absolute value, electrostatic forces repel nanoparticles from each other [22]. Generally, a nanofluid with a measured absolute zeta-potential value above 30 mV is regarded as stable [23]. However, zeta potential results may not be reliable for high salinity base fluids.

(3) Light transmission and scattering method. The intensity of transmitted and scattered light for a single particle is related to the particle volume. Particle size and sedimentation thickness can be measured in real time [8].

(4) Ultraviolet–visible (UV–Vis) spectrophotometry. The absorption of light by nanoparticles is used to calculate the concentration of nanoparticles and quantitatively evaluate the stability of the nanofluid [10].

(5) Scanning electron microscopy (SEM) and transmission electron microscopy (TEM). SEM and TEM are powerful tools to characteristic the shape, size and distribution of nanoparticles. In addition, cryogenic electron microscopy (Cryo-SEM and Cryo-TEM) can observe the real state of nanoparticles in the nanofluid, while SEM and TEM only work for dry samples. [10] Therefore, the aggregation nanoparticles in a nanofluid can be observed directly.

(6) Sedimentation balance method. In this method the tray of a sedimentation balance is immersed in the fresh nanofluid. The weight of nanoparticles sedimentation during a certain period of time is measured, hence the total nanoparticles sedimentation can be calculated accordingly [22].

(7) Three omega method. In this method the stability of the nanofluid can be evaluated by detecting the thermal conductivity growth caused by the nanoparticle sedimentation. Several stability tests refer to this method in the literature. [24,25,26]

For the application of nanofluids in EOR, high temperature, high salinity and the presence of divalent cations such as Mg^2+^, Ca^2+^, and Ba^2+^ present challenges to the colloidal stability. Several literature reports have studied the stability of nanoparticles at reservoir conditions for EOR applications. For example, Metin et al. observed a critical salt concentration for a given salt solution, below which the nanoparticles have a good stability and above which aggregation of nanoparticles occur and sedimentation is observed at the bottom of the samples. They also reported that divalent cations played a more important role in destabilizing nanoparticles than monovalent cations, and that elevated temperatures accelerate the nanoparticle aggregation process [27]. Sofla et al. studied silica nanoparticle stability in synthetic seawater and observed that HCl can effectively stabilize nanoparticles in seawater [15]. Ranka et al. functionalized silica nanoparticles with zwitterionic polymers to undergo a structural transition from a collapsed globule to a more open coil-like structure with increasing ionic strength and temperature. The surface functionalized silica nanoparticles exhibited long-term stability at salinities up to 120 g/L at 90 °C [20]. However, in the aforementioned studies only the effect of salts and temperature on the stability of the nanoparticles was considered. Two other important factors during an EOR process, namely, the effect of reservoir rocks and crude oils, may also play a role in the stability of nanoparticles. In this study several reservoir rocks and crude oil samples from different oil fields were used to investigate their effects on nanoparticle stability under high-temperature and high-salinity conditions. Nanofluid EOR has shown its potential of increased oil recovery in the laboratory by using both coreflood and microfluidic flooding experiments. Hendraningrant et al. has reported that fumed silica nanoparticles could increase oil recovery by 2% to 10% with 20 coreflood experiments [28]. Khezrnejad et al. performed microfluidic flooding experiments with silica nanoparticles and found nanofluid flooding increased oil recovery by more than 10% compared to water flooding [29]. However, these two experiments were conducted under room temperature and used NaCl brine and deionized water, which is favorable for the stability of nanoparticles. This study also tried to find a solution to stabilize silica nanoparticles under reservoir conditions, making silica nanoparticles also have a potential to increase oil recovery for a field application.

## 2. Experimental Materials

### 2.1. Nanoparticle

**Figure 1:**
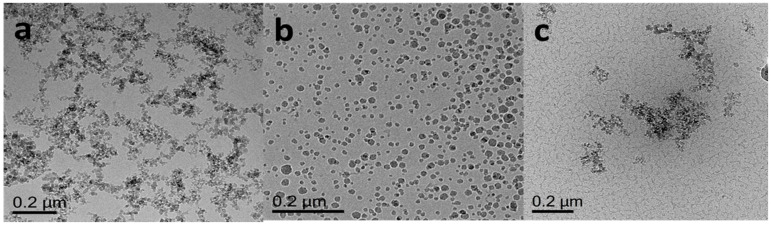
Cryo-transmission electron microscope (TEM) image of nanoparticles: (**a**) fumed hydrophilic silica nanoparticles (FNP); (**b**) amide-functionalized colloidal silica nanoparticles (ANP); (**c**) surface-modified FNP with zwitterionic polymer (FNP-MD).

**Figure 2:**
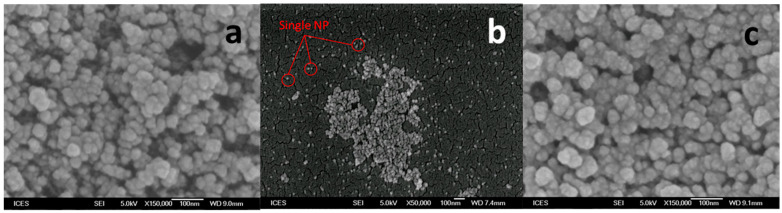
Scanning electron microscope (SEM) image of nanoparticles: (**a**) FNP; (**b**) ANP; (**c**) FNP-MD.

Fumed hydrophilic silica nanoparticles (FNP) were provided by Evonik Industries (Germany). The primary particle size of FNP is about 7 nm, but in suspension nanoparticles will aggregate and the size may increase to more than 100 nm. The specific surface area of FNP is around 300 m^2^/g. Amide-functionalized colloidal silica nanoparticles (ANP) dispersion was purchased from Sigma-Aldrich (Singapore). The particle size of ANP is less than 30 nm. Surface-modified FNP with zwitterionic polymer (FNP-MD) were prepared according to the procedure described in Section 3.4. The nanoparticles were characterized by Cryogenic Transmission Electron Microscopy (Cryo-TEM) (FEI Titan Krios, Thermo Scientific™, Waltham, MA, USA) and Scanning Electron Microscope (SEM) (JSM-6700F, JEOL, Japan). Images are shown in Figure 1 and Figure 2. For Cryo-TEM imaging, the sample (5 µL) was applied onto a grid (Quantifoil, R2/2, Holey carbon film; freshly glow-discharged prior to use at 20 mA for 60 s) without dilution. Excess of sample was blotted away with filter paper to leave a thin film on the grid before being vitrified in liquid ethane. Cryo-TEM measurements were performed on an FEI Titan Krios equipped with automated sample loader and a field-emission gun (FEG) operating at 300 kV. Images were recorded with Falcon II camera (4 × 4) with magnification of 29,000 and pixel size of 2.873 Å [30]. For SEM imaging, diluted nanofluid was placed on a coverslip. After dying, the sample was sputtered with gold and ready for SEM imaging. The high voltage was 5.0 kV, working distance ranged from 7.4 mm to 9.1 mm and secondary electron image was used.

### 2.2. Nanoparticles Suspensions

Five concentrations (0.1, 0.2, 0.3, 0.4 and 0.5 wt. %) of nanoparticles dispersed in lab made synthetic seawater (SSW) of 3.8 wt. % salinity were used. The recipe of SSW is given in Table 1. For FNP and FNP-MD, nanoparticles were weighed and then dispersed in SSW by using a sonicator (40W for 20 min). For HCl-stabilized FNP suspension samples (FNP-HCl), concentrated aqueous HCl was added to the suspension of FNP until pH 2.0.

The ANP suspensions were prepared by a dilution of concentrated ANP dispersion sample. The size of nanoparticles such in suspension was measured using ZetaSizer Nano dynamic light scattering (DLS) (Malvern Panalytical, Malvern, UK) in a standard 12 mm polystyrene cuvette. The samples was measured at 25 °C without any dilution at 173º backscatter [30]. The measured particle size for FNP, FNP-MD and ANP are 142.5 nm, 168.3 nm and 21.4 nm, respectively.

### 2.3. Reservoir Rocks

Two Berea sandstones (BSS) (Kocurek Industries, Inc, Caldwell, TX, US) with low (BSS1) and high (BSS2) permeability were used. Chalk, limestone and shale were also used in this study. The mineral composition (by X-ray diffraction) for these rock samples are shown in Table 2. Pure quartz sands (Sigma-Aldrich, Singapore) were used as well for control experiments.

### 2.4. Oils

Decane and eight different types of crude oils (CO1-7) were used in this experiment. Crude oil properties were measured and are given in Table 3.

## 3. Experimental Methods

### 3.1. Nanoparticles Suspension Stability

Nanoparticle suspension stability tests were performed by using visual observation and turbidity scanning. For each type of nanoparticle (FNP, FNP-HCl, FNP-MD and ANP), samples with five different concentrations (0.1, 0.2, 0.3, 0.4 and 0.5 wt. %) were prepared and put into test tubes, which were then placed into a heating cabinet at 70 °C. Photographs of all test tubes were taken after certain period of time to show the stability of samples over time. A Turbiscan LAB colloidal stability analyzer (Formulaction Inc., Toulouse, France) was used to quantify nanoparticle suspension stability. Samples of nanoparticle suspension were scanned at 60 °C with an 880 nm near-infrared light-emitting diode (LED) source and the transmission and backscattered signals were registered by detectors. Since nanoparticle size can affect these signals, delta transmission and backscattering were used to determine nanoparticle stability. The dimensionless turbidity scan index (TSI) defined by the Turbiscan software (TurbiSoft Lab, 2.2.0.82-2, Toulouse, France) was used to quantify nanoparticles suspension stability. Some samples need longer scanning time. They were scanned continuously for the first 10 days then were taken out of the instrument and put into the heating cabinet with a temperature of 60 °C. They were placed back for a single scan every second day. Calculation of *TSI* is based on comparing each scan to the previous one for the selected height and dividing the result by the total selected height in order to obtain a result which does not depend on the quantity of product in the measuring cell. The lower the *TSI* value, the better is the stability of the sample. The following equation was used to calculate the *TSI* [31].

(2) $$TSI=\underset{i}{\sum }\frac{\sum_{h}\left|scan_{i}\left(h\right)-scan_{i-1}\left(h\right)\right|}{H}$$

### 3.2. Effect of Reservoir Rocks and Crude Oils on Nanoparticle Stability

FNP, FNP-MD and ANP suspensions at 0.1 wt. % concentration were used in this stability test. The nanoparticle suspension (7 mL) was put into a test tube and 0.2 gm of crushed rock sample was added. Pure quartz was used as a benchmark for the rock samples. Another set of samples was prepared with 5 mL nanoparticle suspension and 2 mL oil in test tubes. Decane was used as reference oil. The test tubes were placed inside a heated cabinet at 70 °C.

### 3.3. Particles Size and pH Measurement of Nanoparticles

The particle size distribution of the nanofluids was measured by DLS until nanoparticles were fully agglomerated. The pH of nanofluids was measured with a pH meter.

### 3.4. Preparation of Polymer Modified Nanoparticles

[2-(Methacryloyloxy)ethyl]dimethyl-(3-sulfopropyl) ammonium hydroxide (MEDSAH, 95%, Aldrich, Singapore), 2-(2-carboxyethylsulfanylthiocarbonylsulfanyl)propionic acid (CTA, 95%, Aldrich, Singapore), 3-(trimethoxysilyl) propyl methacrylate (MPS, 98%, Aldrich, Singapore), 2,2′-azobis(2-methylpropionamidine) dihydrochloride (V50, Wako Chemicals, Osaka, Japan), fumed silica (FNP), toluene (high-performance liquid chromatography (HPLC), VWR Chemicals) were used as received.

**Figure 3:**
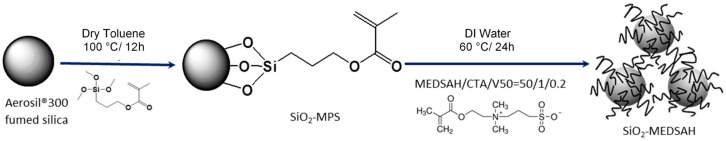
Schematic of preparation polymer modified nanoparticles.

In a sealed Schlenk flask, fumed silica was dried under vacuum at 120 °C for at least 24 h before being used. As illustrated schematically in Figure 3, to a dispersion of dried silica (2 g) in toluene (100 mL) being stirred (1000 rpm) and under argon protection, MPS (3 g) was added using an argon-purged syringe. The mixture was then heated to 100 °C for 12 h. After cooling to room temperature, the dispersion was centrifuged and washed with fresh toluene (3 times), followed by ethanol rinsing (3 times). The methacrylate-functionalized silica (SiO_2_-MPS) was dried under vacuum at 60 °C for 12 h. As-prepared SiO_2_-MPS (0.5 g) was dispersed in deionized water (25 mL) under sonication for 2 h. MEDSAH (2 g), CTA (36.6 mg), V50 (7.8 mg) (MEDSAH/CTA/V50 = 50/1/0.2) were placed into a dry Schlenk flask. The flask was sealed with a rubber septum and subjected to 4 vacuum/argon cycles. SiO_2_-MPS dispersion was deoxygenated by bubbling argon for about 1 h and added to the Schlenk flask using an argon-purged syringe. The mixture was heated at 60 °C under a stirring rate of 700 rpm. After 24 h, the dispersion was cooled to room temperature, and washed with deionized water for 8 times. The polymer-coated silica (SiO_2_-MEDSAH) was dried under vacuum at 80 °C for 24 h.

## 4. Results and Discussion

### 4.1. Nanoparticle Suspension Stability Tests

Four types of nanoparticle suspensions (FNP, FNP-MD, ANP and FNP-HCl) were used in the stability tests. The stability of nanofluids was determined by visual observation and quantified by turbidity scanning analysis. The time when nanoparticle agglomeration occurred for each sample was recorded and is given in Table 4. It can be seen that the unmodified fumed silica (FNP) suspensions had the worst stability. All samples agglomerated and settled within one day. The polymer modified fumed silica (FNP-MD) suspensions had the best stability. All five samples with different concentrations were still stable after thirty days. Adding HCl to fumed silica (FNP-HCl) delayed nanoparticle agglomeration. For fumed silica suspensions, stability increased with decreasing nanoparticle concentration. However, ANP colloidal suspensions showed the opposite trend. The reason is that acid was used to stabilize concentrated ANP suspension by manufacturer, so that 0.5 wt. % diluted sample (pH 4.1) had a lower pH than the 0.1 wt. % diluted sample (pH 5.3).

**Figure 4:**
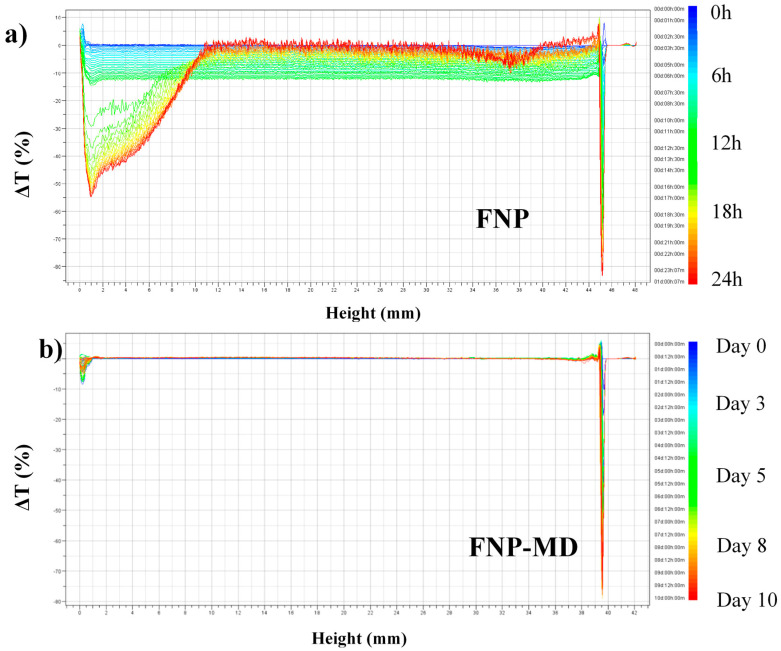
Delta transmission scanning results of nanofluid samples. (**a**): FNP (for one day); (**b**): FNP-MD (for 10 days).

The turbidity scanning tests were performed for nanoparticle suspensions of 0.1 wt. % concentration at 60 °C (instrument limit). As an example, Figure 4 shows the delta transmission (difference of transmission single strength between subsequent scans and initial scan) scanning results for FNP and FNP-MD samples, in which FNP was scanned for one day and FNP-MD was scanned for 10 days continuously. In Figure 4, the blue curve indicates the initial scan and the red curve indicates the final scan. The X axis indicates the sample height (left = bottom of the vial) and the Y axis indicates the delta transmission single strength (in %). As shown in Figure 4a, the FNP nanofluid underwent a strong change in stability. Between 0 h to around 14 h (blue to green), the transmission single strength reduced slightly, which showed that nanoparticle agglomeration occurred and average particle size increased, but nanoparticles were still suspended. However, when the particle size increases to a critical value where gravity dominates, the sedimentation of nanoparticles occurs. In Figure 4a, this phenomenon happened after around 14:30 h and a marked change of delta transmission curves was observed. With more nanoparticle sedimentation, the transmission single strength declined strongly at the bottom of the FNP sample. On the contrary, due to clarification the transmission single strength in the middle and top of the sample increased back to the initial value. In Figure 4b, no significant change of transmission single strength was observed for polymer modified FNP nanofluid over ten days, indicating that it has a good stability compared with the unmodified FNP nanofluid. This result also showed that after surface modification the repulsion force was strong enough to keep nanoparticles apart from each other, so that no agglomeration was detected over ten days.

**Figure 5:**
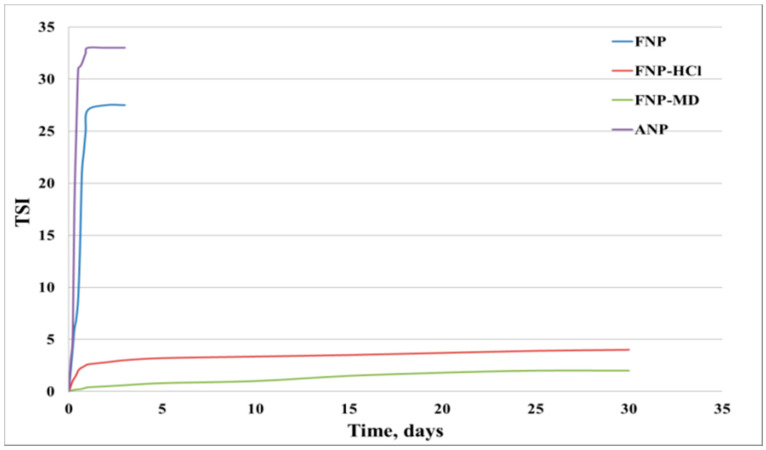
Turbidity scan index (TSI) results for nanoparticles suspensions.

The TSI values of the samples were calculated based on transmission scanning results and plotted versus time (Figure 5). It can be seen that both FNP-HCl and FNP-MD had relatively low TSI values over 30 days, which means that these two samples had a good stability. However, TSI values for FNP and ANP samples increased very fast to a plateau and stayed constant after one day, which indicated that the nanoparticles had agglomerated, settled and lost their stability. This result is consistent with test tube observations.

### 4.2. Effect of Reservoir Rocks on Nanoparticle Stability

**Figure 6:**
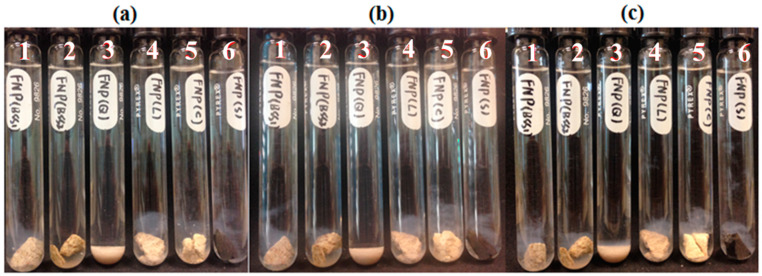
FNP suspensions with reservoir rocks: (**a**), Day 0; (**b**), Day 1; (**c**), Day 3. (1: BBS1; 2: BSS2; 3: quartz; 4: limestone; 5: chalk; 6: shale).

**Figure 7:**
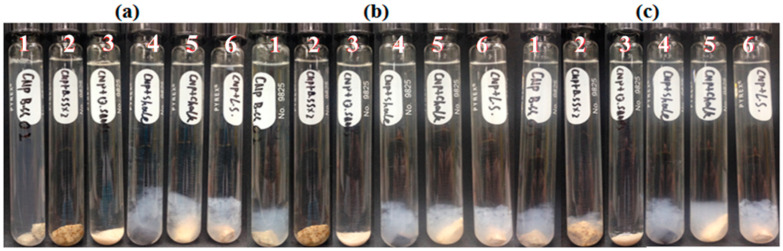
ANP suspensions with reservoir rocks: (**a**), Day 0; (**b**), Day 1; (**c**), Day 3. (1: BBS1; 2: BSS2; 3: quartz; 4: shale; 5: chalk; 6: limestone).

**Figure 8:**
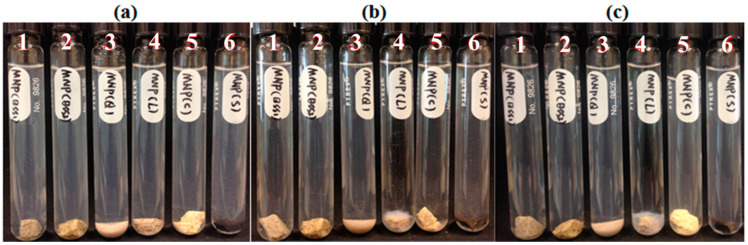
FNP-MD suspensions with reservoir rocks: (**a**), Day 0; (**b**), Day 7; (**c**), Day 30. (1: BBS1; 2: BSS2; 3: quartz; 4: limestone; 5: chalk; 6: shale).

**Figure 9:**
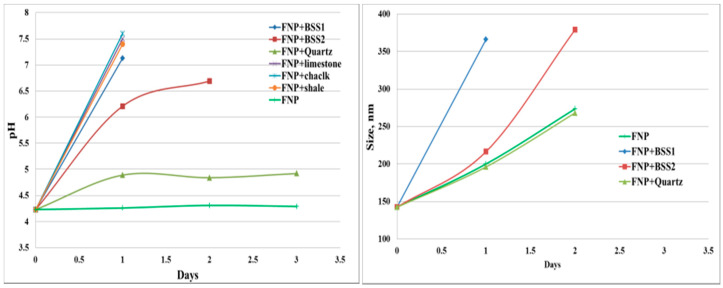
pH and particle size measurements for FNP suspension with different rocks.

**Figure 10:**
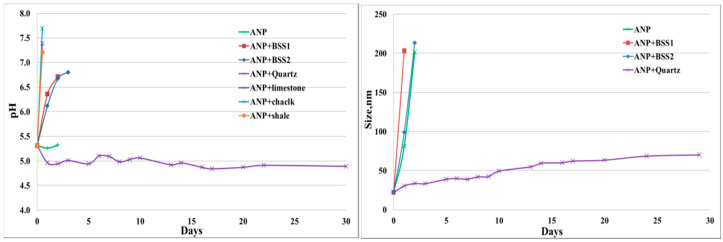
pH and particle size measurements for ANP suspension with different rocks.

**Figure 11:**
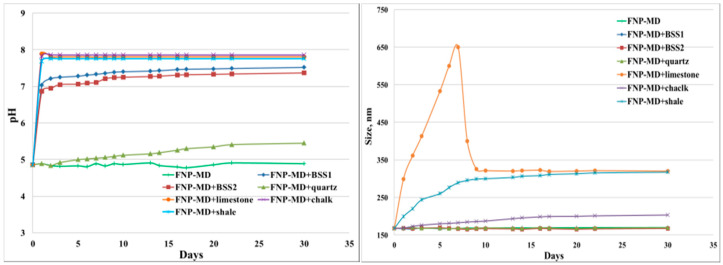
pH and particle size measurements for FNP-MD suspension with different rocks.

Five reservoir rocks (Berea sandstone 1 and 2, chalk, limestone and shale), as well as pure quartz sand as a control mineral, were used in this experiment. FNP, FNP-MD and ANP suspensions with 0.1 wt. % concentration were used. Photographs of all samples in test tubes were taken for nanoparticle stability observation. Particle size and pH of nanoparticle suspensions were measured to investigate the mechanisms for stabilization or destabilization of nanoparticles due to the presence of rock samples. The photographs for FNP, FNP-MD and ANP suspensions with rock samples are shown in Figure 6, Figure 7 and Figure 8. The results of pH and particle size measurements are shown in Figure 9, Figure 10 and Figure 11. As evident from Figure 6 and Figure 7, reservoir rocks had a significant effect on the stability of FNP and ANP, especially for limestone, chalk and shale. These three rocks have very high calcite content (see Table 2), so they could react with H^+^ in the nanoparticle suspensions. In Figure 9 and Figure 10, the pH value of limestone, chalk and shale samples increased to pH 7 quickly, and this neutral pH is not favorable for nanoparticle stability. Thus, fast nanoparticle agglomeration was observed. The Berea sandstones also had an effect on FNP and ANP suspension stability. Nanoparticle agglomeration was observed for BSS1 earlier than for BSS2. The reason might be the higher dolomite content in BSS1 (Table 2), which leads to a quicker pH increase of the suspensions, therefore the nanoparticle size in BSS1 samples increased faster than in BSS2 samples (Figure 9 and Figure 10). No significant effect of quartz sands on FNP stability was observed, while quartz sands stabilized ANP for longer time (Figure 7) compared with the stability result of ANP without quartz sands shown in Table 4; and particle size increased much slower than others (Figure 10). The reason for this phenomenon is unknown and needs further study. For FNP-MD suspensions (Figure 8), there was almost no effect of the rocks on the nanofluid stability, except for limestone. As shown in Figure 11, the pH increased in all reservoir rock samples, but FNP-MD nanofluids were still stable after 30 days in the heating cabinet, even though the particle size increased in chalk and shale samples. For some unknown reason, limestone destabilized the FNP-MD suspension. Since limestone has a similar mineral composition like chalk, more tests need to be done to determine the mechanism of this phenomenon.

### 4.3. Effect of Crude Oils on Nanoparticle Stability

**Figure 12:**
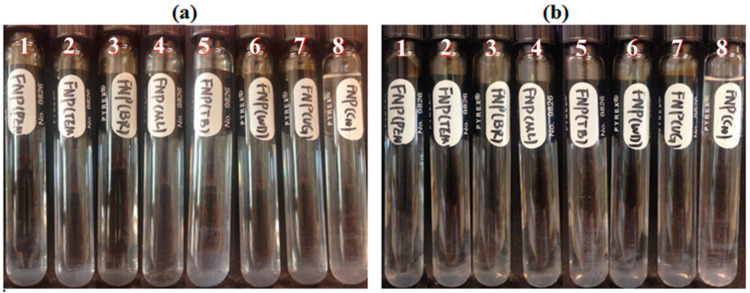
FNP suspensions stability screening with different type of oils: (**a**), Day 1; (**b**), Day 2; (1: CO1; 2: CO2; 3: CO3; 4: CO4; 5: CO5; 6: CO7; 7: CO6; 8: Decane).

**Figure 13:**
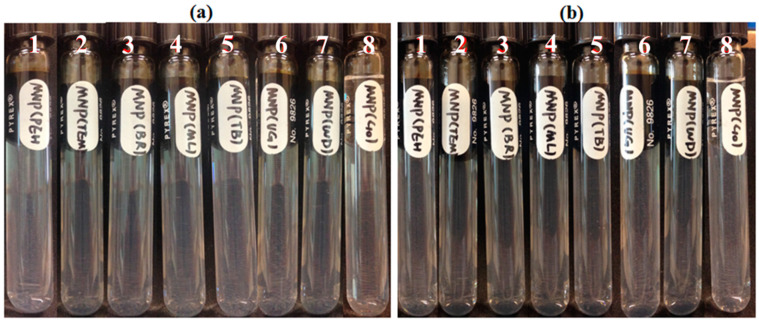
FNP-MD suspensions stability screening with different type of oils: (**a**), Day 1; (**b**), Day 30; (1: CO1; 2: CO2; 3: CO3; 4: CO4; 5: CO5; 6: CO6; 7: CO7; 8: Decane).

**Figure 14:**
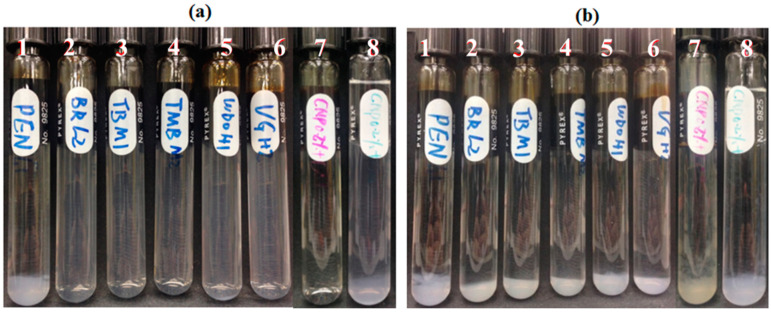
ANP suspensions stability screening with different type of oils: (**a**), Day 1; (**b**), Day 3; (1: CO1; 2: CO3; 3: CO5; 4: CO2; 5: CO7; 6: CO6; 7: CO4; 8: Decane).

Seven crude oils with different properties were used in this study, decane was used as a control oil. FNP, FNP-MD and ANP suspensions with 0.1 wt. % concentration were used. Photographs of all samples in test tubes were taken for nanoparticle stability observation. For FNP nanofluids (Figure 12), crude oils had no obvious effect on their stability. Only nanoparticle agglomeration delay was observed in the CO4 sample on the first day. FNP-MD showed a good stability in the presence of crude oils and was still stable after 30 days in the heating cabinet (Figure 13). For ANP suspension, nanoparticle sedimentation occurred in CO1 and decane samples on day 1, and all samples aggregated on day 3 (Figure 14). For samples with CO2, CO3, CO4 and CO5, nanoparticle sedimentation appeared yellowish. This might be due to migration of some components of the crude oil into the aqueous suspension which adsorbed on the nanoparticle surface, which indicates that crude oil has the potential to affect the structure of nanoparticles in suspension.

## 5. Conclusions

Stability screening tests were performed for a range of unmodified and surface-modified silica nanoparticles, as well as silica nanoparticles with HCl as stabilizer by using methods of stability visualization and turbidity scanning. The results showed that surface modification with polymer and addition of HCl can improve stability of fumed silica nanoparticles under high-temperature and high-salinity conditions remarkably. The turbidity scanning method is a quite useful technique for nanoparticle stability studies, it can reveal nanoparticle agglomeration and sedimentation processes and also quantify nanoparticle stability. The effect of reservoir rock samples and crude oils on nanoparticle stability was also examined. It was found that chalk, limestone and shale destabilized fumed silica nanoparticles and colloidal silica nanoparticles quickly, while they had no obvious influence on the stability of polymer-modified fumed silica nanoparticles. It was also observed that the Berea sandstone containing more dolomite destabilized fumed silica nanoparticles and colloidal silica nanoparticles faster than low dolomite content Berea sandstone. The mechanism of these destabilizations is probably the reaction between carbonate minerals and H^+^ of the nanofluid, leading to a pH increase that accelerated the agglomeration of nanoparticles. However, polymer-modified fumed silica nanoparticles showed a good long-term stability. Quartz sands could stabilize colloidal silica nanoparticle longer than the blank stability test without quartz sands in aqueous suspension. Crude oils had limited effect on fumed silica nanoparticle and colloidal silica nanoparticle stability and no obvious trend was observed, only agglomeration delay was observed in a few samples. Some components of crude oil migrated into the colloidal silica nanoparticle suspensions. Polymer-modified fumed silica nanoparticles had a good stability in the presence of crude oil over 30 days.

## Figures and Tables

**Table 1:** Synthetic seawater recipe.

Salts	Concentration (g/L)	Salts	Concentration (g/L)
CaCl_2_·2H_2_O	1.76	Na_2_SO_4_	4.81
MgCl_2_·6H_2_O	11.23	NaCl	27.03

**Table 2:** Mineral components of rock samples.

%	BSS1	BSS2	Chalk	Limestone	Shale
SiO_2_ Quartz	90.87	90.31	--	0.69	2.84
Na AlSi_3_O_8_ Albite	0.89	1.89	--	--	--
KAlSiO_8_ Sanidine	0.46	0.74	--	--	--
KAL_2_(Si_3_Al)O_10_(OH,F)_2_ Muscovite	3.75	3.6	--	--	2.07
Al_2_SiO_5_(OH)_4_ Kaolinite	1.92	2.29	--	--	1.48
(Mg,Fe)_5_Al(Si_3_Al)O_10_(OH)_8_ Clinochlore	0.97	0.89	--	--	--
CaMg(CO_3_)_2_ Dolomite	1.13	0.28	--	--	1.89
CaCO_3_ Calcite	--	--	100	99.31	89.28
Ca_5_(PO4)_3_(OH)_2_ Apatite	--	--	--	--	2.44

**Table 3:** Crude oil properties.

	CO1	CO2	CO3	CO4	CO5	CO6	CO7
Saturates	60.66	75.72	84.61	38.48	74.79	26.37	32.94
Aromatics	10.52	17.43	12.76	56.13	19.79	51.90	52.26
Resins	9.68	3.85	2.32	5.33	5.06	20.76	14.80
Asphaltenes	19.15	3.00	0.31	0.07	0.36	1.09	0.00
Density@70 °C (kg/m^3^)	826	793	786	852	782	926	902
Viscosity@70 °C (mPa·s)	7.66	2.47	1.90	2.43	1.51	35.61	13.06
Sulphur Content (ppm)	657	491	655	1300	574	3325	1900
Total Acid Number (mg/mg KOH)	0.064	0.475	0.103	0.260	0.239	1.720	1.702

**Table 4:** Nanoparticles stability screening test.

	Days Until Agglomeration Was Observed			
NPs Conc. (wt. %)	FNP	FNP-MD	FNP-HCl	ANP
0.1	1	--	--	1
0.2	1	--	25	8
0.3	<1	--	15	25
0.4	<1	--	12	--
0.5	<1	--	10	--

--: no nanoparticles agglomeration was observed within 30 days.
